# Presumed septic sacroiliitis in a puppy with unilateral hind limb lameness and sciatic nerve neuropathy

**DOI:** 10.1002/vms3.519

**Published:** 2021-05-09

**Authors:** Derniese Goh, Arthur House

**Affiliations:** ^1^ Peninsula Emergency and Referral Hospital Mornington Vic. Australia

**Keywords:** infectious arthritis, joint disease, sacroiliac joint, sacroiliitis, septic arthritis

## Abstract

A 5‐month‐old female entire Dachshund presented with an acute onset of left hind limb lameness following intense play. There were concurrent intermittent neurological deficits in the left hind limb, and pain in the lumbosacral region and on dorsal flexion of the tail. Computed tomography (CT) imaging revealed an asymmetric widening of the left sacroiliac joint with loss of cortical margins, accompanied by mild osteolytic changes of the adjacent ilium and sacrum highly suggestive of septic sacroiliitis. There was also perilesional steatitis in the region of the sciatic nerve. An 8‐week course of antimicrobial therapy was prescribed. Complete resolution of all clinical signs was noted at a 7‐month follow up. Sacroiliitis should be considered as a differential for unilateral hind limb lameness with or without intermittent sciatic neuropathy in a puppy in the absence of other orthopaedic conditions.

## Graphical abstract

This is a case of presumed septic sacroiliitis in a puppy presenting with unilateral hind limb lameness and sciatic nerve neuropathy. There was complete resolution of clinical signs and return to normal function following a course of broad‐spectrum antibiotics.

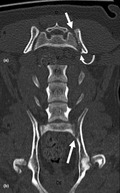

## 1 INTRODUCTION

The sacroiliac joint is composed of a synchondrosis component craniodorsally, and a synovial component caudoventrally. The osseous surfaces of the synchondrosis component are uneven and variably concave, and the synovial component is smooth. The sacroiliac joint connects to the wings of the ilium and the sacrum and is stabilised by dorsal and ventral sacroiliac ligaments. The ventral branches of spinal nerves L6 to S2 travel along the ventral aspect of the sacroiliac joint to form the lumbosacral plexus (Evans & de Lahunta, 2013). The function of the sacroiliac joint is to support the caudal portion of the body, and transmit propulsive forces from the hind limbs to the spine (Breit & Kunzel, 2001). The synovial part of the joint allows for rotation and translation of the ilium relative to the sacrum. The synchondrosis part stabilises the joint, and together with the sacroiliac ligaments, restrict craniocaudal and dorsoventral movement of the ilium relative to the sacrum (Breit & Kunzel, 2001).

Septic arthropathy of the sacroiliac joint is rare in dogs and is not well described in the veterinary literature. In humans, sacroiliitis is uncommon and can be caused by various aetiologies including trauma, infection, degenerative disorders and inflammatory processes (Kanna et al., 2019). Non‐specific physical examination findings may make achieving a diagnosis difficult and thus delay appropriate treatment. The most common presenting sign is pain localised to the lumbosacral region. Radiographic studies of the caudal spine and hind limbs are often normal in the early stages of the disease. Advanced imaging such as a CT scan or Magnetic Resonance Imaging (MRI) are required to evaluate the sacroiliac joint.

This case report describes the diagnosis of presumed septic sacroiliitis including pertinent imaging findings, treatment and prognosis in a puppy.

## 2 CASE HISTORY

A 5‐month‐old female entire Dachshund presented with an acute 4‐day history of back pain and difficulty rising on her hind limbs. She was known to be an extremely active and energetic dog. Three days prior to presentation to her regular veterinarian she was running and playing enthusiastically at the beach. The following day it was noted she was reluctant to exercise and collapsed in her hind limbs whenever she attempted to stand. She was up to date with vaccinations and anti‐parasitic preventatives and had no prior medical history. She was subsequently referred to the surgical department for further investigation.

On physical examination, there was a mild left hind limb lameness and a mild pain response on flexion of her tail dorsally. There were also equivocal neurological abnormalities in her left hind. There were no other abnormalities detected on examination.

**FIGURE 1:**
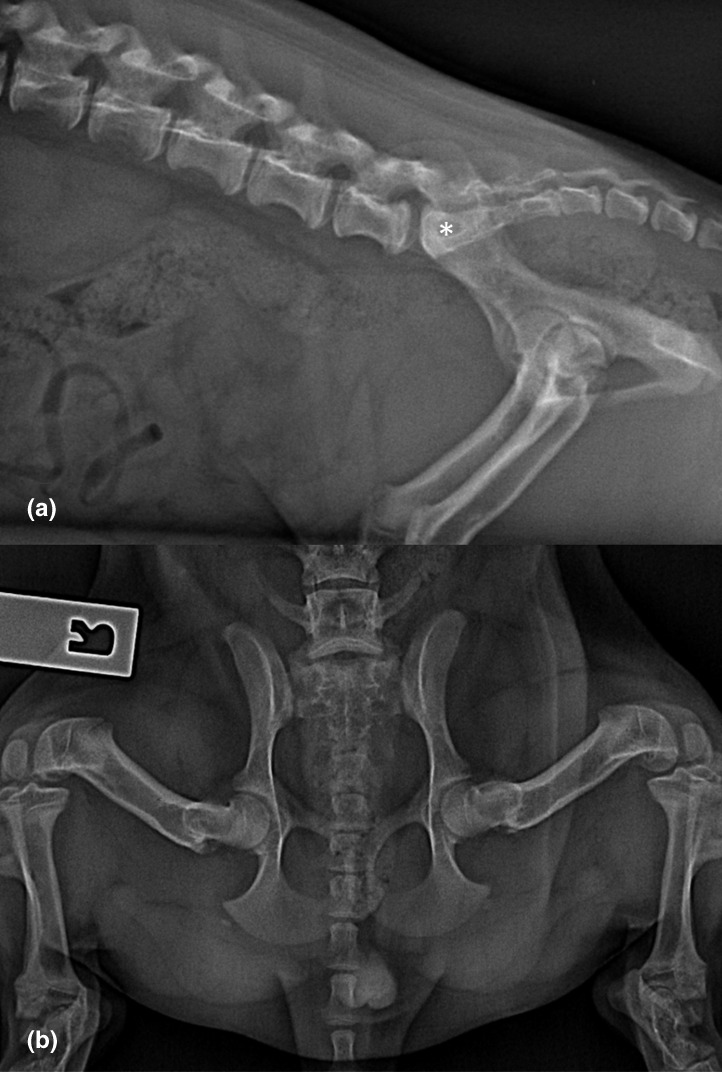
(a) A lateral radiographic projection of the caudal lumbar spine and pelvis. The caudal lumbar and sacrum (*) appear to be radiographically normal in this projection. (b) A ventrodorsal radiographic projection of the sacroiliac joint and pelvis. There is subtle loss of well‐defined cortical margins in the left sacroiliac joint (arrow) as compared to the contralateral joint

Survey radiographs were obtained under general anaesthesia. A lateral projection of the lumbar spine including the hip joints and the proximal coccygeal region, mediolateral and caudocranial projections of the hocks and stifles, and a hip extended and a splayed ventrodorsal projection of the hip joints were performed. On the hip extended ventrodorsal radiographic view (Figure 1), the margins of the left sacroiliac joint were subtly irregular and less well defined as compared to the right sacroiliac joint. The remainder of the radiographs were consistent with a skeletally immature dog and there were no other abnormalities identified (Figure 1). Additionally, the hip joints were assessed for laxity using the Ortolani test with the dog in left and right lateral recumbency respectively (Ortolani, 1976). The hip joints were assessed for luxation in both craniodorsal and caudoventral directions. The tests were negative, and subluxation was not able to be induced. It was elected to trial a short course of Meloxicam 0.1 mg/kg (Metacam; Boehringer Ingelheim) per os once daily for a week.

There was a mild response observed initially in terms of her pain levels. However, she quickly re‐presented a few days later with deterioration in clinical signs and worsening of pain. She remained otherwise systemically well in herself. On presentation she was non‐weight bearing lame on the left hind limb and struggled to ambulate. There was lumbosacral pain and pain on extension of the left hip joint. She had developed a hunched posture in her lower back. Apart from pain on palpation of the lumbosacral region, there were no other apparent neurological deficits.

**FIGURE 2:**
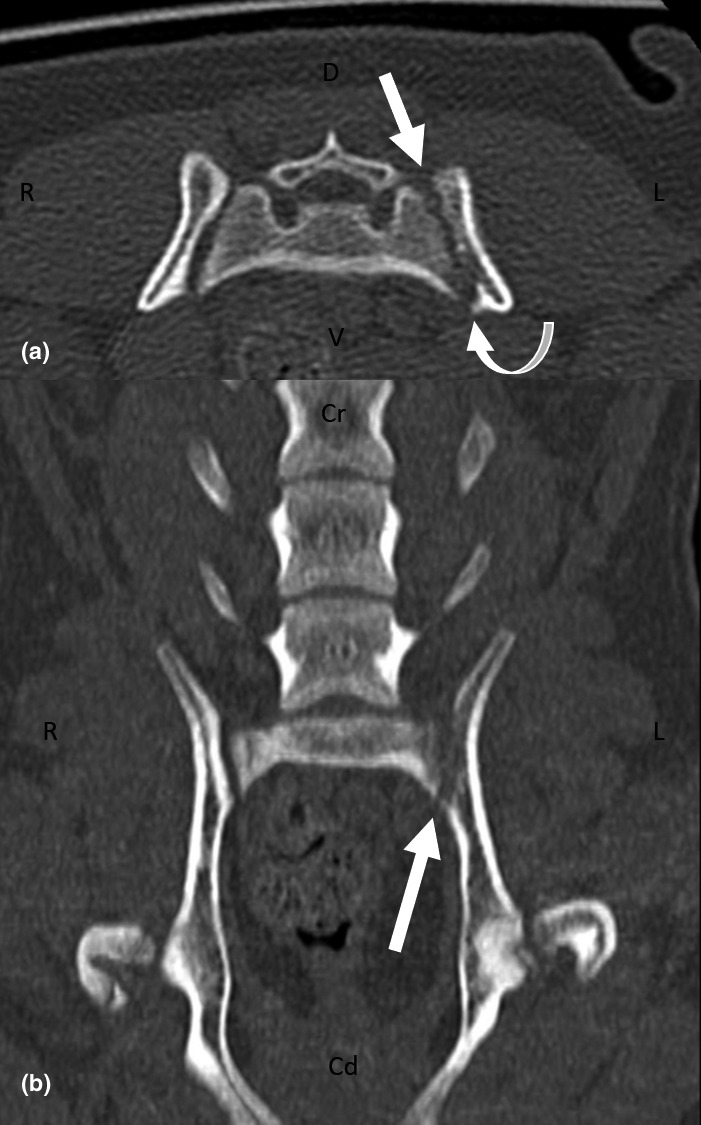
A transverse (a) and dorsal (b) multiplanar reconstruction CT image at the level of the sacroiliac joint using bone windowing (window width, WW = 2,500, window level, WL = 500). There is asymmetric widening of the left sacroiliac joint with an irregular loss of sharp cortical margins with evidence of mild bony destruction of the ilium, and to a lesser extent, the lateral aspect of the sacrum (block arrows). There is subtle amorphous periosteal proliferation of the left ilium immediately caudal to the sacroiliac joint (curved arrow). Cd, caudal; Cr, cranial; D, dorsal; L, left; R, right; V, ventral

**FIGURE 3:**
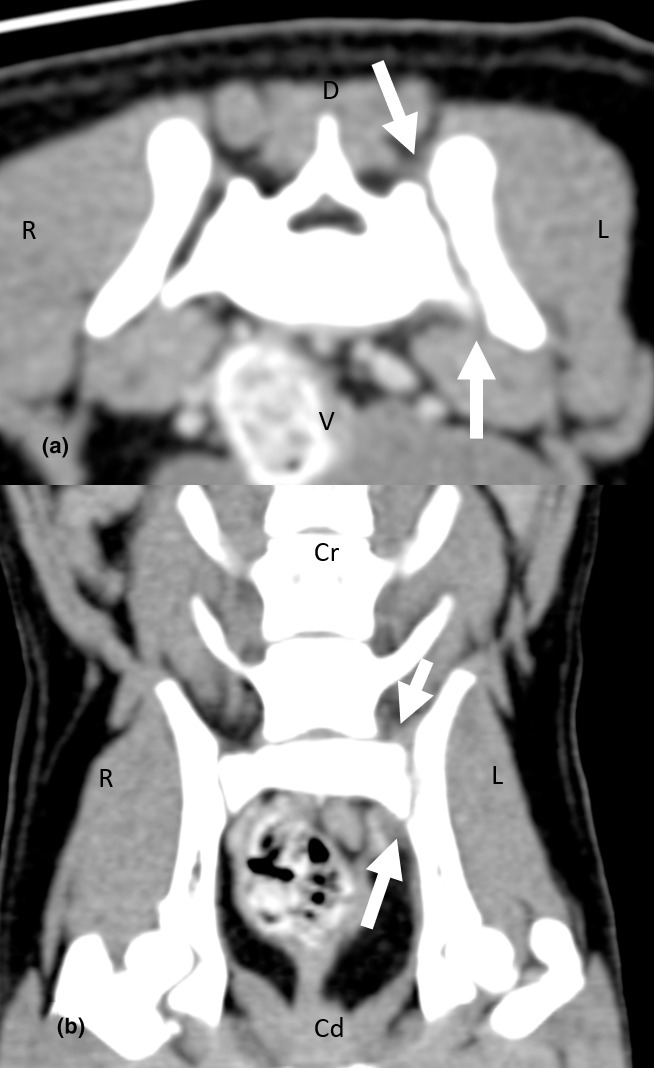
A post‐intravenous contrast transverse (a) and dorsal (b) multiplanar reconstruction CT image of the sacroiliac joint using soft tissue windowing (window width, WW = 320, window length, WL = 40). There is contrast enhancement of the soft tissue immediately adjacent to the left sacroiliac joint (arrow). Cd, caudal; Cr, cranial; D, dorsal; L, left; R, right; V, ventral

**FIGURE 4:**
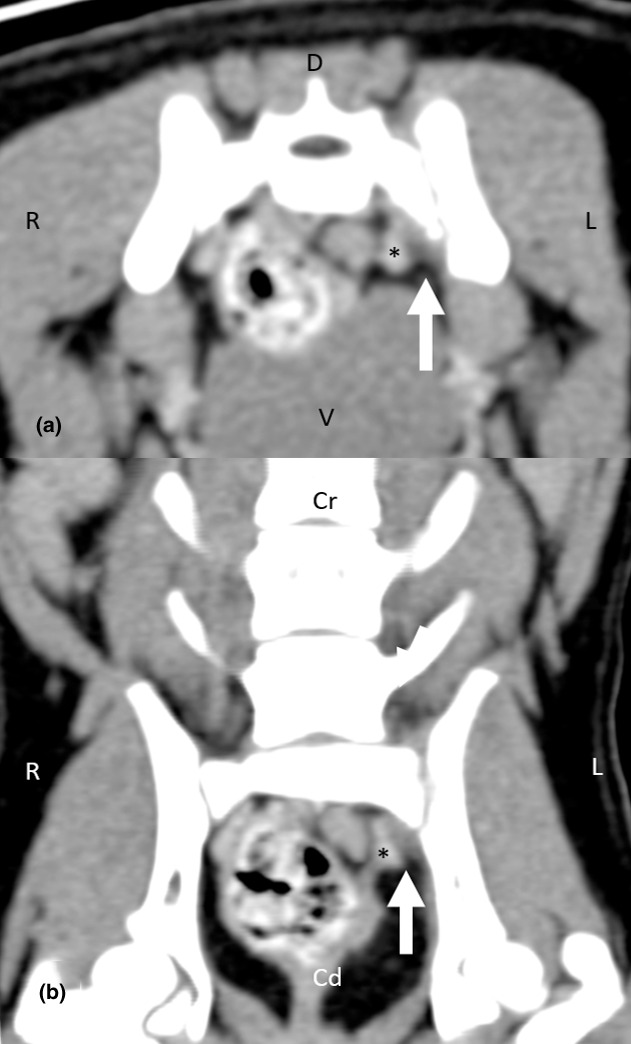
A post‐intravenous contrast transverse (a) and dorsal (b) multiplanar reconstruction CT image of the sacroiliac joint using soft tissue windowing (window width, WW = 320, window length, WL = 40). There is evidence of left sacroiliac joint swelling, and perilesional steatitis and fat stranding (arrow) in the region of the left sciatic nerve and nearby blood vessel (*). As a result, the sciatic nerve and blood vessel (*) are being displaced medially. Cd, caudal; Cr, cranial; D, dorsal; L, left; R, right; V, ventral

A CT scan (GE Healthcare LightSpeed 16 Slice CT Scanner) of the lumbar vertebrae and hind limbs were performed with the patient under general anaesthesia. Imaging parameters were 0.625 mm slice thickness, 100 kVp, 200 mAs, 15 cm field of view, and reconstructed using bone and soft tissue algorithms. Imaging of the tarsi, stifles and hips were done with the patient positioned in sternal recumbency with both hind limbs extended. Imaging of the lumbosacral joint was done with the patient positioned in sternal recumbency with both hind limbs extended in the initial series, then with both hind limbs flexed in the subsequent series. Pre‐ and post‐intravenous contrast iohexol 2 ml/kg (Omnipaque 300 mg I/kg; GE Healthcare) studies were carried out. The images were interpreted by a board‐certified radiologist. There was asymmetric widening of the left sacroiliac joint with an irregular loss of sharp cortical margins. Immediately adjacent to the left sacroiliac joint, there was mild bony destruction of the ilium, and to a lesser extent, the lateral aspect of the sacrum (Figure 2). There was subtle amorphous periosteal proliferation of the left ilium immediately caudal to the sacroiliac joint (Figure 2). In addition, there was perilesional steatitis and fat stranding, particularly in the region of the course of the left sciatic nerve (Figures 3 and 4). Based on the CT findings, the main differential diagnosis included inflammation of the left sacroiliac joint that was atraumatic in origin, with a concurrent active infection, and evidence of local irritation to the adjacent left sciatic nerve.

Percutaneous arthrocentesis of the left sacroiliac joint was attempted. The patient was positioned in sternal recumbency with the hind limbs in a flexed position. A 22‐gauge 90 mm spinal needle was introduced into the sacroiliac joint via a dorsal approach. Using fluoroscopic guidance, the positioning of the needle was confirmed within the dorsal aspect of the sacroiliac joint. The first sample was collected with gentle suction on the needle using a 3 ml syringe attached to the spinal needle, while the second sample was collected via injection and re‐aspiration of 0.5 ml sterile saline. Cytology analysis of the sample was performed by a board‐certified veterinary pathologist. The sample collected had a low cellular yield and consisted mostly of lipid debris and adipocytes. Bacterial culture of the sample was negative. Haematology and biochemistry were within normal limits. Urinalysis of a sample obtained via cystocentesis was normal, there was no microorganisms and bacterial culture was negative. Arthrocentesis of both stifles and hocks were performed. Cytology analysis of the joint fluid was performed by a board‐certified veterinary pathologist and were reported to be normal with no microorganisms seen.

Treatment was commenced for a potential left sacroiliac septic arthropathy. An 8‐week course of amoxycillin/clavulanic acid 12.5 mg per os twice daily was prescribed, and meloxicam was continued as previously prescribed for another week. It was advised to restrict exercise for 2 weeks. Following commencement of antimicrobial therapy there was an immediate response with improvement of lameness and pain within 12 hr. A 2‐week recheck found no lameness or pain and Meloxicam was discontinued. A revisit a week after the course of antibiotics found no abnormalities on physical examination, and owner reported that she was back to her normal demeanour. A 7‐month follow up found complete resolution of signs with no recurrence and a full return to normal function.

## 3 DISCUSSION

The challenges surrounding a septic arthropathy invariably lies in reaching a definitive diagnosis. The presumptive diagnosis of a bacterial arthropathy was made in this case primarily based on imaging findings, and the drastic improvement with antimicrobial therapy. Furthermore, the worsening of clinical signs despite a course of NSAIDs suggests that the source of the lameness and pain was not solely just inflammatory in origin. Amoxycillin/clavulanic acid was chosen for its broad‐spectrum activity, and its activity against the commonly incriminated bacteria species in a septic arthritis, including *Staphylococcus intermedius*, *Staphylococcus aureus*, and beta‐haemolytic *Streptococci* species (Johnston & Tobias, 2017). *Staphylococcus canis* appears to be the most commonly isolated bacteria in congenital or neonatal spread of septic arthritis (Johnston & Tobias, 2017). Treatment was instituted for 8 weeks, given there were adjacent osseous changes suggesting concurrent osteomyelitis. Due to the inherent anatomy of the sacroiliac joint, a septic arthropathy would expectedly involve an extent of osteomyelitis in the osseous component of the joint. In general, the suspicion of a bacterial septic arthritis can be confirmed based on the typical history and clinical signs, a synovial fluid cytology consistent with a bacterial infection and a positive bacterial culture; often a probable diagnosis of a bacterial septic arthritis can be made based on the first two criteria (Johnston & Tobias, 2017). The sensitivity of synovial fluid culture without incubation in enriched media is reportedly 30%–50% (Clements et al., 2005; Macwilliams & Friedrichs, 2003; Marchevsky & Read, 1999; Scharf et al., 2015). The usage of blood culture bottle incubation increases sensitivity to 50%–80% (Clements et al., 2005; Macwilliams & Friedrichs, 2003; Marchevsky & Read, 1999; Scharf et al., 2015). The lack of a positive culture result should not therefore exclude diagnosis of a septic arthropathy and hence initiation of treatment, especially if sufficient reasons for a septic arthropathy are present as failure to recognize the condition and institute timely treatment may potentially lead to high morbidity such as joint instability or systemic spread of infection.

Septic arthritis involves microbial infection of the synovium and the synovial space. This can occur from haematogenous spread, penetrative injury, surgical trauma or local spread from adjacent tissues. The stifle, elbow and carpus are the most frequently affected joints (Johnston & Tobias, 2017). Majority of cases present as an monoarthropathy. Occasionally, a polyarthropathy may occur in an immature or immunocompromised patient. Puppies may develop bacterial polyarthropathy secondary to omphalophlebitis, streptococcal pharyngitis or uterine or mammary infections in the mother. A congenital or neonatal spread was considered given the young age of this puppy. However, the lack of other systemic signs made this unlikely. Additionally, all other puppies in the same litter, as well as her mother, were reportedly clinically normal with no medical history. Septic sacroiliitis was recently described in a 4‐month‐old male entire Boxer that developed sacroiliitis as a direct extension of an intrapelvic abscess (Slater et al., 2019). This dog also had a concurrent history of chronic diarrhoea refractory to multiple courses of metronidazole therapy. In contrast to the case described here, there were no known risk factors for a septic sacroiliitis. Exercise related trauma to the sacroiliac joint, though not previously documented as a predisposing factor leading to septic arthropathy in dogs, may have played a part in the pathogenesis in this case. This dog was playing vigorously the day prior to the onset of lameness, which could have caused a degree of trauma and inflammation to the sacroiliac joint. It is possible that with inflammation of the sacroiliac joint, there was interrupted tissue integrity and hypoperfusion and reduced host defence mechanisms, which may have led to the subsequent development of a septic sacroiliitis.

The technique of sampling the sacroiliac joint is not well described in the literature. Arthrocentesis of the sacroiliac joint was attempted in this dog however given the sample was non‐conclusive it was elected not to proceed with culture and sensitivity testing. It was possible that the sample was collected from the synchondrosis component of the joint which resulted in a relatively low cellular yield. Ultrasound‐guided sacroiliac injection has been reported to be an effective means to localise and treat pain due to sacroiliac disease in people and horses (Cousty et al., 2008; Pekkafahli et al., 2003). The feasibility and accuracy of an ultrasound‐guided sacroiliac joint injection technique was evaluated in a cadaveric study (Jones et al., 2012). Sonoanatomic landmarks were the L7/S2 articular process joints, iliac wing, sacral wing, sacral lamina and the median sacral crest. The transducer was placed caudally, and the sacroiliac joint space is visualised as a triangular, hypoechoic indentation at the junction between the iliac and sacral wings. A 22 gauge, 40‐ or 60‐mm spinal needle was directed cranioventrolaterally and advanced towards the joint space. The maximum volume that could be injected without resistance was 0.5 ml. While the technique was good for injecting into the synchondrosis portion of the sacroiliac joint, it was fair to poor for injecting into the synovial portion of the sacroiliac joint (Jones et al., 2012). With extrapolation of the results of this study, ultrasound‐guided sampling of the sacroiliac joint via this technique may also be attempted, however sampling of the synovial component of the sacroiliac joint may still prove difficult. CT‐guided sacroiliac joint sampling in people with suspect infection has been described using percutaneous aspiration of the joint, lavage aspiration, bone biopsies of the sacrum, ilium or both, and biopsies of the sacroiliac joint or adjacent soft tissue (Knipp et al., 2018). A posterior or anterior approach to the joint was chosen based on feasibility, technical difficulty and whether the anterior or posterior aspect of the sacroiliac joint was more distended on CT. The overall sensitivity and specificity were reportedly 54% and 86% respectively (Knipp et al., 2018). Aspiration was found to have a higher sensitivity than that of biopsy, followed by lavage aspiration (Knipp et al., 2018). CT‐guided aspiration of the sacroiliac joint in dogs may be explored as an alternative. The accuracy and reliability of sampling of the sacroiliac joint under the guidance of the various imaging modalities, and the sensitivity and specificity of aspiration, lavage aspiration or biopsy have yet to be evaluated in dogs.

Lameness in this dog was attributed to septic sacroiliitis and potential sciatic nerve irritation. Delayed conscious proprioception in the left hind limb had been intermittently observed. Septic sacroiliitis and the surrounding perilesional steatitis likely contributed to a degree of sciatic nerve irritation and hence sporadic neurological deficits. Although screening radiographs performed raised the suspicion of whether the patient's clinical signs were associated with potential pathology in the left sacroiliac joint, these changes were overall subtle, and a diagnosis could not be convincingly made solely based on these findings. Cross‐sectional imaging techniques are the preferred diagnostic imaging modalities to evaluate the lumbosacral region.

CT findings in this dog was similar to that previously reported for sacroiliitis (Forbes et al., 2019). CT scan of a dog with *Brucella canis* sacroiliitis and discospondylitis found variable widening of the sacroiliac joint with adjacent regions of subchondral sclerosis, and regions of decreased cortical delineation adjacent to the sacroiliac articulations, in addition to the changes within several lumbar vertebral end plates consistent with discospondylitis. MRI has also been used to evaluate the sacroiliac joint in a case report involving two dogs (Slater et al., 2019). Sequences acquired included T2‐weighted fast‐spin echo in sagittal and transverse planes, T1‐weighted fast‐spin echo in transverse and sagittal planes, with and without fat saturation, pre‐ and post‐contrast medium administration, dorsal short tau inversion recovery and dorsal T2 IDEAL (Slater et al., 2019). Changes observed include an irregular and widened joint, joint effusion, oedematous bone marrow and periarticular soft tissue structures, and contrast enhancement of the fibrous and cartilaginous aspects of the sacroiliac joint space (Slater et al., 2019).

The main limitation of this case report lies in obtaining a definitive diagnosis of a septic sacroiliitis. Ideally a diagnosis was made on culture results of the sacroiliac joint. Obtaining a diagnostic sample of the sacroiliac joint is shown to be challenging even with fluoroscopic or ultrasonic guidance. Given the rapid response to antimicrobial therapy, repeat sampling or obtaining blood cultures were deemed unnecessary. Repeat CT at follow up may have been useful to document changes to the sacroiliac joint. However, the risk of additional radiation exposure to the puppy, and given the complete resolution of clinical signs, it was determined that repeat imaging was not indicated.

Sacroiliitis should be considered as a differential for unilateral limb lameness, lumbosacral pain and sciatic neuropathy in the affected limb. Heavy exercise may be an inciting cause of sacroiliitis. An extended duration of antimicrobial therapy is recommended, particularly if there are indications of adjacent osteomyelitis on imaging studies. Prognosis appears to be excellent with appropriate treatment.

## CONFLICT OF INTEREST

The authors declare no conflict of interest related to this study.

## AUTHOR CONTRIBUTION

**Derniese Goh:** Conceptualization; Data curation; Formal analysis; Writing‐original draft; Writing‐review & editing. **Arthur House:** Conceptualization; Data curation; Formal analysis; Supervision; Writing‐review & editing.

### PEER REVIEW

The peer review history for this article is available at https://publons.com/publon/10.1002/vms3.519.
